# Integrated Decision Support Systems (IDSS) for Dairy Farming: A Discussion on How to Improve Their Sustained Adoption

**DOI:** 10.3390/ani11072025

**Published:** 2021-07-07

**Authors:** Michel Baldin, Tom Breunig, Roger Cue, Albert De Vries, Mark Doornink, Jan Drevenak, Robert Fourdraine, Regi George, Robert Goodling, Randall Greenfield, Matthew W. Jorgensen, Andy Lenkaitis, Doug Reinemann, Amit Saha, Chakra Sankaraiah, Saleh Shahinfar, Cori Siberski, Kevin M. Wade, Fan Zhang, Liliana Fadul-Pacheco, Steven Wangen, Tadeu E. da Silva, Victor E. Cabrera

**Affiliations:** 1 MILC Group, San Luis Obispo, CA 93405, USA; mbaldin@milcgroup.com; 2 Allflex Livestock Intelligence, Madison, WI 53718, USA; Thomas.Breunig@Merck.com; 3 Department of Animal Science, McGill University, Montréal, QC H9X 3V9, Canada; roger.cue@mcgill.ca (R.C.); kevin.wade@mcgill.ca (K.M.W.); 4 Department of Animal Sciences, University of Florida, Gainesville, FL 32608, USA; devries@ufl.edu; 5 VES-Artex, Chippewa Falls, WI 54729, USA; markd@ves.co; 6 SK Farm Partners s. r. o., Bratislava IV, 841 02 Bratislava, Slovakia; jan.drevenak@skfarm.sk; 7 Dairy Record Management Systems, Raleigh, NC 27603, USA; rhfourdr@ncsu.edu; 8 Nalytix LLC, Hartland, WI 53029, USA; regi.george@nalytix.com; 9 Department of Animal Science, Pennsylvania State University, State College, PA 16802, USA; rcg133@psu.edu; 10 Vita Plus Corporation, Madison, WI 53725, USA; RGreenfield@vitaplus.com; 11 USDA—ARS Livestock Behavior Research Unit, West Lafayette, IN 47907, USA; matthew.jorgensen@usda.gov; 12 GEA Farm Technologies Inc., Naperville, IL 60563, USA; andy.lenkaitis@gea.com; 13 Department of Biological Systems Engineering, University of Wisconsin, Madison, WI 53706, USA; doug.reinemann@wisc.edu; 14 FoGS Global Research and Consultancy Centre, Ahmedabad 382010, Gujarat, India; fogsconsulting@outlook.com; 15 Land O’Lakes Inc., Arden Hills, MN 55126, USA; csankaraiah@landolakes.com; 16 Agriculture Victoria Research, Bundoora, VIC 3083, Australia; shahinfar@uwalumni.com; 17 Department of Animal Science, Iowa State University, Ames, IA 50011, USA; coris@iastate.edu; 18 Department of Animal and Dairy Sciences, University of Wisconsin, Madison, WI 53706, USA; f.zhang@wisc.edu (F.Z.); lfpacheco@wisc.edu (L.F.-P.); srwangen@wisc.edu (S.W.); tdasilva2@wisc.edu (T.E.d.S.)

**Keywords:** data stream, decision-making, integrated decision support tools, prescriptive analytics

## Abstract

**Simple Summary:**

This paper discusses mechanisms to promote the sustained adoption of integrated decision support systems (IDSS) in dairy farming. Integrated DSS are those that receive continuous data streams from on-farm and off-farm data collection systems and integrate them to produce insightful and effective outcomes. Multiple limiting factors are preventing the use and adoption of existing IDSS and could also prevent the adoption of upcoming IDSS. In summary, these factors include value proposition, practical application, ease of use, and data governance issues related to the IDSS. Therefore, awareness and further industry-wide discussion of these components could help in the development and sustained adoption of IDSS in order to facilitate the complex process of decision making. As a commentary paper, the emphasis is on the authors’ extensive experience, expertise, and practical knowledge.

**Abstract:**

Dairy farm decision support systems (DSS) are tools which help dairy farmers to solve complex problems by improving the decision-making processes. In this paper, we are interested in newer generation, integrated DSS (IDSS), which additionally and concurrently: (1) receive continuous data feed from on-farm and off-farm data collection systems and (2) integrate more than one data stream to produce insightful outcomes. The scientific community and the allied dairy community have not been successful in developing, disseminating, and promoting a sustained adoption of IDSS. Thus, this paper identifies barriers to adoption as well as factors that would promote the sustained adoption of IDSS. The main barriers to adoption discussed include perceived lack of a good value proposition, complexities of practical application, and ease of use; and IDSS challenges related to data collection, data standards, data integration, and data shareability. Success in the sustainable adoption of IDSS depends on solving these problems and also addressing intrinsic issues related to the development, maintenance, and functioning of IDSS. There is a need for coordinated action by all the main stakeholders in the dairy sector to realize the potential benefits of IDSS, including all important players in the dairy industry production and distribution chain.

## 1. Introduction

Dairy farm decision support systems or tools (DSS) can be defined as computer information systems that assist dairy farmers to solve complex problems or assist with decision making [1]. In this commentary paper, we are especially interested in newer generation of DSS (integrated DSS; hereinafter referred to as IDSS), which additionally and concurrently: (1) receive continuous live data streams from farm data collection systems (e.g., activity sensors, dairy herd improvement (DHI), feed management, etc.) and potentially data from outside the farm (e.g., climate and weather forecast, geographic information system (GIS), market prices, etc.); and sequentially (2) integrate all these data sources to produce more holistic and insightful outcomes with minimal human intervention and supervision [2]. 

The call to discuss such a new generation of IDSS arises from the fact that we are experiencing a true revolution in dairy farming in terms of the way data are collected and the increasing and ever-growing amount of data generated. Despite the vast amount of data available, data streams can be sparse and disconnected. As such, current DSS are built to address specific problems—they do not present themselves in an integrated way, do not address broader spectrum problems, and do not support data-driven decision-making processes looking at the big picture of the farmer’s needs.

One way to take advantage of this large number of data streams is to apply readily available technologies (e.g., extract, transform, load (ETL) tools, cloud data storage, application programming interfaces (API), data warehouse, etc.) to integrate those different data sources and pipeline them to compose a larger integrated data storage and exchange facility. Only then we can address broader problems and apply the state-of-the-art data science artificial intelligence, modeling, simulation, and optimization techniques to support farmer’s decision-making processes. This is what we conceptualize as IDSS. 

Data sampling covers an enormous range of time scales, from very frequent (e.g., thousands of Hz for some sensors) to less frequent (e.g., monthly herd performance such as a DHI ). The key feature of newer generation IDSS is that they are able to automatically or semi-automatically integrate data sources regardless of their time scale.

To our knowledge, the current use and adoption of traditional DSS by dairy farmers are normally restricted to vendor-specific third party software, applications, or expert systems. It is common that machinery, equipment, or service suppliers deliver their products coupled with proprietary software as is the case of milking parlors, individual cow sensors, or genomic tests companies [3]. Other vendors produce specific software for general herd management, feed management, or financial management. However, with a few exceptions, these applications are mostly focused on narrow and specific problems, therefore not involving any major data integration.

Dairy scientists at the Universities of Florida, Pennsylvania, Wisconsin, and others have produced expert systems that use data extracted from different data farm systems. Additionally, there are some examples of DSS with some data integration include the Dairy Brain [2,4,5,6], a University effort, and various commercial efforts (e.g., MyDairyDashboard.com, Join-Data.nl, Connecterra.io, iDDEN.org, Cargill Dairy Enteligen™, etc.). We judge their development to be restricted to mostly descriptive dashboard applications with limited adoption. 

Some of the envisioned IDSS are not yet available and might not become available until some important barriers are overcome. The scientific community and the allied industry have not been effective or successful in developing, disseminating and promoting a sustained adoption of IDSS for dairy farms [3]. For example, in a recent world-wide review study, only 22 peer-reviewed papers were found to address data integration and their practical application in dairy production decision making [7]. Therefore, there is a pressing need to revisit and discuss basic concepts of DSS adoption even before the envisioned IDSS tools are available to anticipate the best actions that will ensure and enhance uptake and sustained adoption. 

Although dairy farmers are increasingly relying on data and dashboards to make decisions regarding animal and farm management, they are limited to traditional DSS that exist in isolation as an extension of hardware or services implemented on the farm and are not integrated [7]. Our main question is: how can we improve the newer generation of IDSS to be relevant, useful, and remain a critical part of the dairy farm management into the future? 

Incorporation of IDSS as part of routine dairy farm management is paramount for long-term sustainability. For example, IDSS would allow farmers to anticipate disease outbreaks, mange periods of economic hardship or supply shortages, to monitor impacts of diet changes, health programs, or youngstock rearing; to forecast replacement needs, genetic progress, or cropping plans; and optimize reproductive programs, semen type use, genomic testing, and to replacement protocols. All of these management tasks would benefit from continuous data input from multiple sources and are well beyond what is currently available to dairy farmers. The availability of these types of IDSS would almost certainly provide new and valuable insights to improve the management of dairy farms. 

As a commentary paper, the authors’ emphasis in this manuscript was to provide insights after a deep reflection based on experience, expertise, and practical knowledge about what we think would be a pathway for sustained adoption of IDSS. As such, the discussion is not meant to be extensive, definitive, or conclusive, but rather stimulating and provoking, so it can promote an ever larger industry discussion. It is worth mentioning that everything discussed comes from the experience acquired with traditional DSS, since the availability of IDSS is still restricted. 

Thus, our objectives with this commentary paper are two-fold: (1) to identify the main barriers associated with both development and adoption of IDSS, and (2) to discuss some factors we consider fundamental to promote the sustainable adoption of these tools from their conception to their application on farms.

## 2. Background and Procedure 

This commentary paper is the result of a collaborative effort from the University of Wisconsin-Madison Dairy Brain team and the Dairy Brain Coordinate Innovation Network (CIN) (https://dairybrain.wisc.edu/coordinated-innovation-network/). The Dairy Brain CIN is an industry-wide stakeholder group discussing issues of data governance in the dairy industry and guiding the Dairy Brain team in their research and development undertakings. The Dairy Brain CIN was established in September 2019, succeeding an initial project Advisory Committee. Currently it enlists more than 100 members, composed of worldwide dairy industry professionals, policy makers, and academicians, who provide insights and guidance to the University of Wisconsin-Madison Dairy Brain project. 

The Dairy Brain CIN has committed to publish documents about data ownership, data security, best practices for data collection and communication, creating value from farm data, strategies for data utilization and monetization, and related issues. As part of such an effort, the Dairy Brain CIN has already published five articles in Hoard’s Dairyman between February and May 2020 [4,8,9,10,11]. After these publications, the group entertained open forum discussions through its website and social media platforms. These and internal discussions identified those topics with the greatest need for more in depth articles. One of those needs was promoting sustained adoption of IDSS, which this article addresses.

Once the main topic was loosely defined, an invitation was extended to all Dairy Brain team and all CIN members to gather their interest and willingness to collaborate and co-author this manuscript. Listed authors of this article are the ones who responded positively to the call and contributed throughout the process. 

An outlined draft document was shared among all participants and remained open for edition, contribution, suggestions, and commentary during the entire process. The process involved discussion focus groups, weekly meetings with a few selected Dairy Brain members, constant communication between the Dairy Brain CIN and the Dairy Brain team, and prominent feedback among all the participants. The Dairy Brain team took the lead on processing and updating the manuscript, but the whole group had the opportunity to input into the process at any time. A highly inclusive environment was promoted to ensure a truly collaborative effort that represents the consensual opinions of all the authors.

## 3. Factors Affecting Adoption of IDSS

Adoption of IDSS is dependent on its value proposition, opportunity cost, trust, and time constraints. From the farmers’ point of view, they might not see the potential value proposition of a possible IDSS adoption. Farmers might be wary of the investment required for an IDSS adoption. Farmers might not have the interest or the incentive to take the risk of adopting a new IDSS. Many risks with respect to adopting a new IDSS could exist. One risk is that the data streams will not remain integrated in the future and therefore the IDSS would become obsolete. Another risk is that the IDSS value would result in being not greater than its price investment. Still another risk is the uncertainty about having proper maintenance, service, and customer support for the tool. The tools may be perceived as not addressing pressing and important needs on the farm. Or farmers simply might not have the time to investigate the potential of IDSS. It could also be that infrastructure such as adequate internet connectivity, equipment, facilities, or knowledgeable personnel are lacking. From the IDSS’s side, they could be too expensive, not meet the farmer’s expectation, or lack adequate robustness to significantly impact decision making.

### 3.1. Perceived Added Value to the Farm

Dairy farmers are focused on finding alternative ways to increase profitability of their business and therefore are concerned about finding the value proposition of each decision they make. In the case of IDSS, the value proposition is not always readily apparent. Behind IDSS, there could be a large number of sophisticated calculations and algorithms that sometimes display outcomes that might not always be intuitive in the eyes of farmers. Complexity of the calculations per se is not the main barrier, but how the farmer perceives the results is. For example, most dairy producers trust genetic evaluations even though they might not understand all the underlying calculations and algorithms. Dairy producers know, by experience, that they can improve genetic progress on their herds by selecting superior sires to be used in their reproduction programs—the value is apparent through improvements in milk production, conformation, and health in subsequent generations of cattle. The clear presentation of a possible value proposition, whether it is that the IDSS would help to solve a relevant problem or that the IDSS would support optimal decision making, is paramount in terms of uptaking and sustaining an adoption.

The value proposition of an IDSS could be the single most important factor for adoption. Recommendations from IDSS might not be trusted initially because they point to a future situation that is difficult to envision, verify, or replicate, which may take considerable time to realize the benefits. In this sense, depending on the IDSS proposal, it is essential to make it very clear to the end user the IDSS expected outcomes—if the IDSS’s recommendations are for decision making in the short (operational), medium (tactical) and long (strategic) term. Therefore, farmers and other decision makers would face the choice to invest time and money on a new IDSS for which would have returns with a previously aligned period of time after the investment has been made. It could also happen that an IDSS determine better performance, but the improvement might not be worth the investment. For operational or tactical tools, the return on investment can be answered in a relatively short period of time, whereas it would take years for strategic ones. Furthermore, complexity in the farm operation and the increasingly integrated nature of the dairy sector would make it difficult to determine if improvements can be attributed to the IDSS. 

Take as an example the case of farmers selecting semen from top bulls and compare it with proposed culling policy decisions. Both could come from IDSS, both would require sophisticated algorithms for calculation, and both would require years to display its full benefits. The main difference could be that farmers would likely trust the long-standing genetic selection system and would feel comfortable with its “intangible,” more intuitive benefits of genetic progress, in the long term without a need for much proof. For the case of the culling policy, they would likely need more evidence, which sometimes can only be achieved with time through a process that follows the maturity of the technology.

Without waiting for performance evaluations long into the future, farmers and practitioners might use as a proxy the fact of how well the IDSS represents the farm currently. If the IDSS does not represent the farm well, it would be a clear reason for the farmer to distrust the tool. Perhaps the best situation would happen when the tool is capable of representing the farm relatively well and still be able to propose improvements that are reasonable in the mind of the decision makers—accurately outlining the current situation on-farm, as well as demonstrating how the proposed decisions could provide value into the future. Understanding potential value (the value proposition) will be much easier in situations wherein the IDSS tool generates trust from the producer, and this may be the most critical aspect of future adoption.

In many cases, the value proposition can be summarized simply as bottom line increased profit. Farmers would be very vocal in recognizing that unless they truly believe that the tool will allow them to make more money, they would not consider implementing it. Regardless of the management area, whether it is health, fertility, or nutrition, the tool should address the main issues preventing them from being more efficient and more profitable. Therefore, studying with the farmers in those areas for “profit opportunity” could be a good place to start the development of tools that will be relevant. This also implies that, to have a measurable impact on decision making, the IDSS to cause an impact on the farmer would need to have tangible values attributed to the proposed improvements. There might be situations where the decision is not directly associated with net returns such as the case of compliance with environmental regulations. We believe that even in those situations, economic impact is central to producer decision making. 

It is also important to recognize that farms are very different and are at different levels of management; therefore, the tools would have inherently different values to different farms. As an example, if a tool to help farmers decrease somatic cell count (SCC) is proposed, that could be good news and would probably be rapidly adopted by farms having >400,000 cells/mL, but not by those farms that are already consistently having <100,000 cells/mL. The latter farms would have minimal room for improvement in their bottom line when further decreasing SCC count, so they would likely have a different interest in the tool, such as maintenance of a low SCC and prevention of potential losses related to SCC level increases. 

A tool would also have a different impact at different levels among farms and even within the same farm. It is important that an IDSS is developed with a clear understanding of the specific target audience who would likely benefit from it. For example, if we rank the producer population from top to bottom regarding performance, modernization, or technology availability, it is necessary to ask ourselves which group of farmers the tool is designed for? Similar differences have to be recognized in multi-unit dairy operations, which are becoming more common around the globe. These multi-unit dairy operations would comprise several milking parlors, facilities, and fields and might even outsource some parts of their operations, all of which brings a greater level of complexity in needs with respect to IDSS and data. These multi-unit operations might also be more interested in performance comparisons among units rather than with other farms.

The possible added value of an IDSS is intrinsically related to the needs or wants of farmers or end users. It is widely recognized that before starting to build an IDSS, an effort should be devoted to understanding those needs. Proper need assessment involves not only listening carefully to farmers, but also understanding all challenges farmers face and devising solutions that would go beyond what farmers even knew would be possible. Farmers who are presented with a tool that addresses pressing needs are more likely to consider adoption. Such a proposition, though, could have the issue of concentrating more or only on current issues rather than looking at the big picture and on analyzing the whole integrated system in the long run. Paying attention only to perceived needs could miss innovative opportunities that go beyond what is recognized by the farmer or managers. Indeed, the nature of the IDSS makes them especially interesting to discover new and perhaps better operating pathways. 

IDSS are also envisioned as vehicles to control unintended consequences that could occur in one farm management area when changes are performed in a different, and perhaps distant, management area. A combination of needs assessment, participatory tool development, and end user engagement together with scientific innovation and sophisticated algorithms, could be optimal to develop IDSS that are not only relevant in the eyes of the users, but also that generate innovative solutions while minimizing potential negative outcomes. 

As with any innovation, there would be pioneers (early adopters), followers (which are the majority and follow the pioneers peer in a moderate phase) and laggards (slow adopters or skeptical, which will take them a long time to adopt any new ideas, if at all), so peer communication and reinforcement play an important role in adoption, which should be recognized when communicating the value proposition of the IDSS. It is important to mention that innovation in dairy farms is not always related to farm size. Although it could be assumed that larger farmers are more likely to try and adopt faster, small farmers can be as innovative as their larger counterparts. Hence, regardless of farm size or farmer profile, peer farmer recommendation could be the most effective and powerful way to promote and maintain IDSS adoption.

Although the final adopter is the farmer or farm decision maker, the input of the farm’s consultant or advisor would hold a large weight in the adoption decision. It is not uncommon that the farmer will call the consultant(s) (e.g., nutritionists, financial advisers, veterinarians, etc.) before making an adoption decision to ask them about the possible benefits in the production system and, importantly, their tool’s perceived value proposition. In a certain way, consultants become the gatekeepers as they would hold great leverage on the adoption decision. Tools should also facilitate and not interfere with the consultant’s job or role on the farm. Therefore, an important target public, as IDSS are being envisioned and developed, are farm consultants who should also be involved in the whole adoption process. Consultants could be persuaded to take it as an opportunity to have one more tool in their toolbox and therefore to have better possibilities to support and engage with the farmer through tool discussions. In the end, IDSS should support not only farmers, but also consultants’ decision making. 

The lack of perceived value proposition can have several causes and it may not be solely related to the IDSS per se. For example, farmer’s education level or experience will play a role in their ability to make data-driven decisions and maybe on perceived value. From this perspective, with the underlying assumption that a specific IDSS is valuable to the farm, training could be an alternative to improve perceived value proposition and as a consequence adoption and sustained usage and application. Training is also important to guide the farmer to the most relevant indicators in the production system and how to make better decisions based on them using IDSS. 

Wide and multiple touches of dissemination together with a “champion on site” to promote the tool will likely make or break the adoption. Also worth mentioning is the fact that tools do not need to be heavily sophisticated to be useful and adopted. Sometimes simple outcomes from IDSS like feed efficiency or milk income over feed cost monitoring systems (which although simple in their calculation, require continuous data integration streams), could end up being very helpful and heavily adopted by farmers. At the end of the day, IDSS must deliver insightful outcomes for decision-making processes based on the most holistic assessment possible of their production system.

### 3.2. Easiness to Use and/or to Interpret

Understanding and adapting the IDSS to provide the best experience to the user is crucial. If the tools are difficult to use and/or difficult to interpret, there may be issues with their design. We need to keep in mind that the target audience of the IDSS are the farmers and hence the development process should be user-centered. Therefore, we need to develop a good understanding of farmers’ profiles as IDSS users. An IDSS that has not been designed according to its target audience or does not address its audience’s needs is likely to fail. The concept of user experience encompasses all the users’ emotions, beliefs, preferences, perceptions, physical and psychological responses, behaviors, and accomplishments that occur before, during, and after use of the IDSS. 

One way to achieve a good user experience is to have the user participating in the whole development of the IDSS. The process could begin with a proof of concept in which the user can visualize the concept idea and already provide feedback. Repeating the process of making changes and seeking feedback means that development in all phases could be iterative, which at the end will promote a tool with greater accessibility and thus greater adoption. 

The benefit of many tools is not realized until there is efficacious support available to help the end-user understand the tool’s functionality and the interpretation of the outcomes. Here, again, consulting or training may be critical, as having a professional working with the producer in the adoption process can filter better ideas and solidify the tool’s efficacy. This implies a lot of interaction between the farmer and the IDSS team and other potential stakeholders such as consultants or industry professionals. Maybe there is a need to figure out a system in which there is a social element attached to the participative platform that accompanies the farmers throughout the process of adoption. Social interaction becomes a justification to revisit the tool constantly, which would help farmers realize and internalize some intangible value or long-term return on investment that is not easily seen or understood. An important action could, for example, be producers discussing the IDSS as a group, which could potentially lead to a better tool and greater and sustained adoption. 

In addition to application-related factors and development, usability and output interpretation may be improved with a good training program. This can be a key factor not only to guide farmers and managers to make a better use of the IDSS, but also to get permanent feedback and a better understanding about their user experience. All feedback needs to be on a list of priorities, subsequently evaluated by the technical and development teams, and then included in a continuous improvement process pipeline.

The more intuitive the tool, the better the adoption. In general, users prefer tools that are more visual, intuitive and simple. It is good to train farmers and consultants on the use of the tool, but it may send the wrong signal if the tool needs too much training prior to independent use by the producer themselves. The tool interface also dictates a big part of adoption success. Modern farmers and other users are used to having apps at their fingertips that are extremely intuitive and easy to use. Producers are often hesitant to use tools with complex interfaces, or which require complex coding or commands, so IDSS should heavily limit such situations.

Also related to interface simplicity is interface appearance; many of the tools still being used by farmers look completely outdated by today’s standards, malfunction frequently, and are incompatible with newer computing systems. On the other extreme, we can name examples of tools that can be very sophisticated and potentially very useful, but they do not reach the end users due to their complexity. Take as an example tools related to mitigation of price risks. In the US and other countries, price risk management tools exist for dairy farmers who can hedge their milk and feed prices, if so desired. Use of these tools could protect against unexpected market variability but, nonetheless, these are only marginally used at best. Perhaps potential users might not completely understand them or do not feel comfortable using them. The tool value may only be apparent in bad situations, and producers may perceive hedging to be a costly proposition under normal conditions or prevent maximum margins under best market conditions. These types of tools are heavily related to the user’s personal risk preferences. For example, risk averse farmers would be more likely to consider them. Thus, risk aversion preferences should also be considered and even incorporated in IDSS. 

### 3.3. Practical and Direct Application

Irrelevant outcomes without direct link to farmers’ needs may reduce IDSS’s perceived value. In general, from its conception, any IDSS should be meaningful enough in order to address important issues faced by the farmers and then generate reliable outcomes to guide the decision making needed to reach a solution. Furthermore, whatever is developed as an IDSS needs to have a clear deliverable action plan on the farm and/or something that can be directly applied. Perception of significant value may also serve to reduce hesitance in other obstacles to widespread adoption—if users see great value in the final tool, other possible concerns such as privacy or security might be reduced or eliminated. Tool value and applicability should outweigh the potential additional concerns.

Maybe the “carrot” of the adoption lies in straightforward messages such as alerts that are simple and direct, regardless of all the data and algorithms behind them. Even more, IDSS should not only show the alert, but also propose possible actions or solutions to the situation. You have the farmer’s attention if the tool is able to show in a dashboard the decrease in feed efficiency in a pen, predict what would be needed as feed resources the next month, or suggest breeding protocols to attain maximum genetic progress and profit with only raising the required replacements. On the other hand, we have to be cautious not to overload the farmer with all kinds of alerts all the time. Alerts need to be responsive to important events or trends and at the same time they should disregard trivial events or trends. Good IDSS require the right balance of sensitivity and specificity to provide an acceptable level of true positives and true negatives. Additionally, farmers should have the opportunity to adjust the level of responsiveness of the tool according to their preferences. This is especially important when there is a division of responsibilities on the farm and the decision-making process is not centralized to a single individual but is dispersed among others who make up the farm team. 

Farmers have multiple tasks to do during extremely busy days so systems that provide them alerts of only the most important issues are likely to be more useful, appreciated, and maintained. They would be better served if they receive specific and targeted alerts. For example: “trends show that the milk urea N is going up,” which is prompted before a small issue becomes a big problem and there is yet an opportunity to reverse the course. An ideal system would be the one that provides to the farmer a ranked list of the top issues to attend to on this day, this week, this month, this year, etc. A smart IDSS environment should act to make life easier for the producer, not act as a timesuck or overburden with unneeded information.

Lack of practical application has a direct impact on adoption and perception of added value. Tools that provide some outcomes that need to be pre-processed or included in other applications before they can actually be acted upon might encounter adoption issues. This is especially important on IDSS that rely on several data sources to perform properly. In practical terms, this is something that can take a long time and be subject to errors which would generate resistance and greatly undermine the possible adoption. Here, our initial concept of “integrated” DSS is critically important because it implies that not only the algorithms, but also the data preparation and transfers are all being taken care of behind scenes, without the need of the farmer’s intervention or supervision.

A large number of farms seem to struggle with transforming raw data into actionable information or even noting the potential that raw data have when processed into something usable. We might all agree that if we do not measure something we cannot improve it—this is especially true for farmers. However, it is not that simple. The important action to accurately record data would not magically convert the data into actionable information. The data must become information, and the information needs to become action. Tools can help to visualize, summarize, compare, analyze, etc., but they are not useful if they do not end in an effective and positive action. 

Taking into consideration that producers have very different levels of maturity in terms of decision making based on data and differ greatly according to the availability and amount of data they generate, manage, or have access to, it would be desirable to design tools that can be usable at each of these levels. Integrated DSS could have different levels of possible outputs in concordance with the potential users and their available resources, reflecting the farm size, finances, and end-user ability. This would depend on the needs of the producer, the level of applied technology and the level of maturity. At the end, the practical and direct application will depend on how meaningful the IDSS are according to the final user in front of the tool. Trustability of the IDSS information would also be dependent of how much the data have been transformed from their original source. Farmers may be more inclined to trust the information if the data have been barely manipulated or it is still easily traceable to their original source. Farmers may have a harder time relating to data that have gone through an involved process of transformation, which become an additional barrier in the adoption process.

One other important point about IDSS is the fact that they need to be adaptable to ever changing situations. We do not know what is going to happen 10 years from now regarding new technologies in the farm and how they can all be leveraged to produce even higher level or better decisions. Tools can become obsolete with time. An example would be the use of an IDSS to improve health status in the herd. If the use and application become efficient, the farm might achieve a desired level of health, which is either optimal or below which there is no additional economic benefit and therefore the farmer has no additional incentive to keep improving by using the tool. At that point, the tool could become a monitoring system to keep herd health status under control across time. The tool could be used within a set of standard operating procedures to ensure desired levels are under acceptable levels over time. In addition, as novel health-related farm practices, breeding plans, and monitoring tools become available, the tool can integrate these factors into future decision matrices to ensure continued maximum profitability. For adoption to be sustained over time, tools need to be nimble and responsive to both internal and external changes relevant to the user base. 

For instance, fast adaptability could mean the development of well-documented systems in a prioritized modular fashion, with agile architecture and infrastructure design, that can be easily reprogrammed to incorporate new technologies as they are being developed. If the development of IDSS is fast and successful, evolving technologies could even follow the tools’ platform. However, that would also depend on the specific vendors and their own commercial interests. At the very minimum, we should try to anticipate and create IDSS that are flexible enough in their design and would not need to be redone from scratch to accommodate emerging technologies. We suggest being open to constant feedback from the end users and other stakeholders. In this regard, we may reinforce the need to have a “champion on site”, who can act as a bridge between the needs of the end user and the IDSS development team (i.e., dairy specialists, financial specialists, programmers, etc.). 

A challenge is to design tools that could be tailored to farm-specific use. For example, a tool could alert the possible onset of cow’s clinical mastitis based on a number of data sources connected continuously (e.g., milk harvested, milk flow, milk conductivity, genomic data, and historical health records). However, in many cases, the farmer may not have all of this information available to feed the IDSS. The question that arises then is, how to keep the tool relevant, even when presented with a less robust data stream. Given that we are talking about data-dependent tools, the viable path seems to be to develop a defined hierarchy of resources for decision making which would allow the tool to take advantage of what data actually are available in the farm. Obviously, the level of sophistication and power in terms of decision making will increase proportionally with the availability of data, but a fully informed model cannot be the assumed starting point. It is likely that, as the farmer considers all the possible benefits of working with a fully informed IDSS, this would promote more and better data collection standards on that farm, further improving the tool’s capacity to effectively support decision-making processes within the farm. We are not advocating for the development of extremely tailored IDSS to meet specific needs and situations. This is not something desirable because IDSS should be general enough to cover a large number of decision-makers, but at the same time they should be able to adjust and tailor based on farm-specific data situations.

### 3.4. Data Collection Standards and Data Quality

Data quality is an important issue for IDSS development and adoption. Although dairy farm systems generate vast amounts of data, sometimes the quality of data is poor. Trying to build high-quality models with poor data happens in reality (i.e., garbage in, garbage out). There are large amounts of data available on dairy farms which include daily milk production, milk composition (e.g., protein, fat, lactose, and somatic cells count), electric conductivity in milk, activity, rumination, temperature, estrus detection, health, genetics, and much more. Data quality is a core concern in terms of decision-making processes based on IDSS because dairy farmers will only rely on tools that have ensured reliability based upon data quality. However, it is worth emphasizing that data quality is a shared responsibility and the farmer is an essential part of ensuring this. Improving and maintaining data quality is an integral part of the data collection process for the IDSS to work properly. 

Closely related to data quality, data standardization is a major challenge in the dairy industry. The same factor may be referred to by several different names, there are different ways to calculate what is supposed to be the same thing, and there are some measurements that are not only farm, or unit specific, but even employee specific (e.g., body condition scoring). The first step to address these shared vocabularies issues would be to encourage the dairy community to adopt a more universal and standardized collection of dairy concepts, terms, definitions, and relationships [e.g., Food and Agriculture Organization (FAO/Agrovo ontology)—fao.org/agrovoc]. Similarly, it would be opportune to adopt any standard and established guidelines for best practices regarding both dairy data collection and calculation of productive and reproductive indicators [e.g., International Committee for Animal Recording (ICAR)—ICAR.org ]. Although these data can be cleaned manually for research purposes, it is very difficult to set a consistent protocol of rules that would process different sources of data continuously with minimal or no supervision. Data issues in general, whether they are about quality, standardization, or integration are especially important for IDSS as these tools rely completely on those data.

Different technologies could provide the same measurement differently, which erodes our abilities to build models and IDSS. As an example, rumination data are very specific to the technology attached and the measurement of this behavior differs between type and brand of sensor used in measurement. Given this situation, the use of those data in a IDSS cannot be blind to the data origin and its particularities. Additionally, the issue of accuracy and data collection frequency play an increasingly important role. Take as another example the case of cow-level fat content in milk measurement, which could be very accurate but sparse in frequency on a monthly basis in the DHI tests. Now, compare this with a high frequency system such as an in-line sensor which measures fat content in every milking, which however might have lower accuracy. Our development of IDSS should take into account all the available technologies and propose the best alternative options. Following the examples above, it would be required that the IDSS be able to optimally use all available data in a particular farm and leverage all the options of data frequency and accuracy. Furthermore, tools should be malleable and responsive to ever-changing conditions such as an improvement in the accuracy of the sensor measurements. Considerations in our above example include at which accuracy level in-line sensors can replace the monthly DHI test, or, perhaps which combination of sensor data and DHI test data is optimal for decision making.

There are a number of initiatives and large investments in the industry that have promoted greater and more frequent data collection that unfortunately are not being fully utilized. There are large repositories of data already in existence. It may be more efficient to invest in building an infrastructure that allows the data to flow better towards an envisioned IDSS. Typically, great ideas and effort which go into building good tools become overshadowed by the enormity of the effort of simply bringing the data together readying it for the IDSS to use. The proposition value of the data is what you do with it, regardless of how much effort you put into collecting, cleaning, and standardizing, tasks which normally occur behind the scenes and eventually require more effort than developing the actual IDSS. Hence, any proposition able to devote greater consideration to the actual development of the data integration for the tools is likely to have greater chances of success. The challenge remains on how to deal with the data from the collection point to the tools ingestion point. In our experience, once we got permission from the farmer to access the data the major bottleneck has been, and continues to be, the effort of readying the data ready for analysis. For this to happen, we need a greater level of data standardization. Organisms like ICAR (ICAR.org) have been defining those standards for years and some of those have been adopted by National Dairy Herd Improvement Association, Quality Certification Services (iDDEN.org), etc.

It is likely that some farmers would be reluctant to enter into an agreement in which they are expected to share all their data blindly for IDSS development and use. Farmers likely need to know which data are being used in which tool and for what purpose. The farmers need to feel in control of their data, including how their data flows from their farm to the systems, how it is finally utilized, and how their privacy is protected in this process. This is important now and likely will become even more important in the future—the growth of technology implementation in dairy means that both the number of stakeholders and size and number of data sources to track will only increase. Even a dashboard of data flow will be an important IDSS development tool that will provide transparency in the data sharing process.

Having ready-to-use IDSS should not stop farmers from further collecting data and improving data collection quality and standards. It is often perceived by users that they can ‘plug-and-play’ to the IDSS indefinitely. However, it is important to bring to our attention that the farm management, environment, genetics of animals, and a number of other factors are constantly evolving. Therefore, accurate and detailed data collection for re-training and updating IDSS will be critical in continued use.

### 3.5. Data Integration

The IDSS is only realistic in an environment of data integration, which involves data collection, data access, data decoding, data cleaning, and data homogenization, for which standardization is paramount. Lack of data standardization and integration might be the most critical bottleneck in the conception, development and execution of IDSS by the technical professionals. There is not currently a robust data connection interface at dairy farms, as happens in other industries. The modern method of sharing data efficiently among data generators at the farm is using application programming interfaces (API), which obviously require data standardization. Most of the applications we use daily in our smartphones, tablets, or computers share data using API as long as we provide permission. Standardization of systems like this is needed and would be welcome in the dairy industry. We need to think carefully about these issues at the conception and during all processes of development of IDSS in the dairy farm industry as these will have a great impact on the final adoption. 

Some technologies adopted by farmers might have multiple applications, but likely farmers will use them only for the original intended use. For example, an activity monitor that measures rumination may also be capable of heat detection simultaneously. If the original intention of the farmer was to measure rumination, it is unlikely that the farmer would take advantage of the heat detection component of the technology. This is important in the adoption process of IDSS because the more efficient a technology, the greater the possible utility, and the more the capacity to interact with other farm technologies, the more likely the value added for the adoption process. Additionally, like in any other technologies, farmers would need to consider new devices or services according to the capabilities of these technologies to share information with other technologies already available on the farm.

### 3.6. Data Sharing and Farm Infrastructure

The industry has to be much more collaborative when considering the current state of data sharing. Private companies serving the industry in any specific aspect from parlors, to feed, or ventilation systems, are unlikely to ever control 100% of the market share. Therefore, there is a need to emphasize and encourage collaboration with other companies, even competitors, in order to provide better service to the farmer. In marketing their product or service, companies send out the message that their system is all that the farmers ever need but, in a rapidly evolving technology ecosystem, new technologies from alternative companies are constantly appearing. Farmers, scientists, and industry professionals are realizing that there is an opportunity to combine data from different sources, hence the big need for mechanisms that allow safe, efficient, and profitable data sharing.

There is increasing consideration of the need for a national approach regarding dairy data collection and handling, for example Lactanet (Canadian Network for Dairy Excellence), which combines data resources into a cohesive effort. However, it appears that other players involved in the generation and collection of dairy related data have different views on sharing the data for the greater good. An example is the trend of “cloud-only” data display protocols in which data storage is not allowed. This increases the risk of limiting the full utilization of all the data generated on farms. The notion is to have a gateway, rather than a database within a mechanism to move data to a portal. These systems become facilitators of data transfer, not aggregators of data. Although restrictive in their conceptual platform, these systems could become reliable networks with established standards, supporting the development and maintenance of these proposed IDSS without the need to ever try to build a gigantic database. Perhaps there would not even be a need for large historical datasets as the memory could reside in the algorithms that sustain the tools.

The potential breadth and depth of available data—and thus quality of subsequent decision making—grows exponentially if an IDSS can incorporate data from other farms, states, or nations. It seems we are at a time in which the industry is reinventing itself on data sharing protocols that challenge the old agreements with a greater emphasis on the value data proposition. Many companies are realizing that they could also make money by selling data or monetizing the data in some fashion. It happens that some companies trying to dominate the market share will push for control; however, it is difficult to imagine one company will control everything within a domain or the possibility that farmers will commit to one single solution provider. 

Although there have been, and are expected to be, large advances in infrastructure, farms are located in remote rural areas and suffer from unreliable internet connectivity, lack of appropriate equipment (e.g., computer and peripherals), technical support, and the other hardware and software taken for granted in other settings. If an IDSS depends on connectivity, a large portion of farms are not yet able to take advantage of its full benefits. Producer access to these resources is a critical consideration in IDSS development and methods to work around such limitations should be taken into consideration in early development, until rural infrastructure receives much needed attention.

As a final stumbling block in IDSS development, important new knowledge and scientific developments with direct implications for farm management are not always accessible to those working on tool creation. This may be due to a lack of meaningful multidisciplinary work between engineers, data scientists, and dairy researchers to connect dairy industry expertise with those capable of constructing and refining measurement systems. These are opportunities missed that otherwise would promote better and sustained adoption of IDSS. It is, for example, useless to apply the most advanced algorithms and build models with high accuracy and precision from datasets that either do not actually represent the features that will be collected on the farm (i.e., are not biologically significant), cannot be connected with other systems at the farm, or do not have the right equipment or technology on the farm.

## 4. Discussion

As demonstrated above, factors that either prevent dairy farmers from adopting IDSS or stimulate them to sustainably adopt the tools, may come from both subjective and objective factors. Farmers and decision makers can become stimulated to adopt and overcome these obstacles when academicians and industry professionals carefully consider factors regarding research, development, and dissemination efforts. There is a need to prioritize the ‘most-wanted features’ for upcoming IDSS based on the existing up-to-date academic outcomes or commercial products. 

Researchers and IDSS developers (both from universities or companies) focus on the ‘state-of-art’ proposed solutions that will be present in the ‘next-generation’ of tools. However, the evolution needs not only adaptation from the final user, but also positive responses reflected from the developers. Ideally, the process should be iterative among all the parties and with continuous feedback loops between users and researchers, so developers can address the ever changing needs expressed by the farmers. Integrated DSS need to be sustainable over time and therefore need to live within a business model in which they can be constantly updated and adapted to the new conditions. This likely requires personnel and facilities. 

The business of developing IDSS lies in bringing additional insights to those that already exist from companies providing services or technologies to farmers. Although the farm has multiple data sources, it is impossible that these come from only one company and therefore there is an opportunity for both companies and farmers to add value to the existing systems on a farm. Take as an example the use of sensors to detect health issues in cows, which could be combined with temperature and humidity collected at the barn, and dry matter intake at the feed bunk to provide a better overall assessment of the herd and individual cow. Such a system could be as attractive to the diverse companies involved as to the farmer in decision making regarding the integrated issues of feeding, environment control, and/or disease management. Here is where more companies would likely have an interest in working collaboratively because no company will have all the data in one place. This proposition would create a great need for collaboration that at the end would be good for the industry overall, a win-win situation for companies and farmers. There is an opportunity and need to build those tools that require multiple data sources (IDSS), which could be enhanced by the fact that there are initiatives and professionals solely interested in the realm of bringing multiple sources of data together to address the real issues facing farmers and who are not competing for selling services or technologies to the farmers. An example is the University of Wisconsin-Madison Dairy Brain project.

To be adopted, IDSS need to have a quantifiable good value proposition to the farmer (e.g., good return on investment). The tools should then provide enough value to the producers, so they can be paid by themselves (through increased production or efficiency, reduced disease, etc.). One important limitation for IDSS adoption could be the financial status of the farm because normally these tools or the data needed for the tool are associated with high upfront costs to install a technology or technology interface. There seems also to be a relationship between farm size, cost, and adoption. Larger farms, with more capital resources and a larger potential return, are likely to try and adopt first. Small dairy farmers might not have the luxury to try new technologies or may receive lower return on investment due to scale. For example, the cost of installing a rumination sensing system (on-animal collars, signal receivers, software, etc.) on a small dairy may not be cost-efficient when the producer can easily walk the barn to monitor the animals without needing the tool. However, it is important to emphasize that the size of the dairy farm should not be an obstacle to the cost associated with the adoption of the IDSS. If the IDSS is built with modular architecture, it is possible to serve the different audiences in view of their different needs. A modular architecture could permit serving dairy farmers with restricted financial conditions, but who want to take advantage of data-driven decisions. Even the best IDSS with a more engaged audience needs to be exposed to its target audience or stakeholders. Then a good marketing and dissemination plan can be a determining factor in the success of the IDSS. 

The cost associated with IDSS has a significant impact on the uptake and adoption by the dairy farmers. Depending on the value proposition, IDSS are more likely to be adopted if they are inexpensive or cost-effective relative to opportunity cost (i.e., other aspects of the farm requiring investment which would be ignored if the IDSS was implemented). Tools need, at the very least, a good on-farm technology of information infrastructure (e.g., computer, internet connection, server, etc.). Besides those, integrated tools require many sources of information and data integration, which need even more technologies collecting data and with those greater budgets for the system. In addition, there are costs associated with maintaining equipment and assembled infrastructure that are taken into consideration with regards to adopting new IDSS. Nonetheless, if the benefits of adopting IDSS are evident and the time and feasibility to return on invested capital are acceptable to the farmer, they are more likely to find a way to make its implementation and maintenance feasible. 

Training could be a good alternative to improve perceived value proposition of a tool and as consequence improved adoption and sustained usage and application. Training is also important to guide the farmer to the most relevant indicators in the production system and how to make better overall decisions based on them. Development, usability and output interpretation may be improved with a good training program, which can play a key function, not only to guide users to make better use of the IDSS. Similarly, frequent contact with producers to gather feedback and address issues adds value over the tool’s lifetime. All feedback needs to be systematized on a list of priorities, subsequently evaluated by the development team and included in a continuous improvement process pipeline. 

Improving the user experience is a fundamental feature. Integrated DSS need to be user-friendly, simple, and intuitive. In general, people are visual learners. Too much text is not desirable. Farmers should see what needs to be seen at a glance. Ideally, IDSS should provide information for decision making anticipating possible problems in the herd. For example, if the percentage of cows with mastitis in the herd went to levels above what is considered normal from one month to the next, this information should be easily taken to the producer. In this sense, instead of placing the producer in front of the computer and making the farmer search for the information, the ideal scenario is for the IDSS to send the information directly in the form of an alert. 

Farmers like to discuss and share information with each other. Recommendation from another peer farmer is probably the most powerful and effective way to spread knowledge and disseminate innovations, which is especially important in IDSS because these are complex products in which concrete outcomes are only envisioned with time. Adoption of IDSS, like any technology, follows a well defined curve of adoption according to different groups and their likelihood to innovate. Early adopters are the innovators that likely drive the adoption of other groups who follow them. A good starting point is to have early adopters as the main ambassadors of the benefits of the tool. Word of mouth, geographical proximity, and community related events in which farmers can see and appreciate each other’s work are paramount in the process of adoption. Integrated DSS could become the vehicle for discussion, interpretation, and feedback in farm peer groups, which promote the tools to be much more valuable and desirable. Thus, if at all possible, keeping in mind features that would allow farmers to interact together related to the tool is a plus in the adoption process. It is worth mentioning that the recommendation by peers will necessarily require a good previous experience with the IDSS. Therefore, everything that has been discussed so far must have been achieved for the peer recommendation to be effective in increasing the level of adoption of the IDSS.

Findings from Rose et al. [7] indicate that the tools ecosystem is far from mature in the agricultural industry. Tools are available and deliverable in a number of convenient formats such as software (for computers), apps (for mobile devices), or traditional paper-based reports. Despite the convenience of these ‘input–output’ formats, farmers still have to manage the utilization of IDSS by themselves. As such, training and understanding of the IDSS functioning are paramount. 

We can make a straightforward forecast that, in a “not too distant” future, the overall number of available IDSS will decrease together with the establishment of international or industrial standards. Old-fashioned tools will disappear and more and more user-friendly, multi-function integrated, modular, and lower-cost tools will become dominant, just as what we have been experiencing in most information technology domains such as computers, smartphones, other software, and apps in general. 

We may not need to “reinvent the wheel.” Addressing conclusions from contemporary studies and comparing them with the up-to-date progress that we are experiencing could be a good approach. This is directly related to how meaningful the tools are, and should be able to respond to the following questions: Is the tool providing results that are directly applicable? Is the tool using several data sources available at the farm? Are outcomes integrated with other tools? Is the tool a unique need for a specific producer or is it an industry shared need? 

One strong aspect that affects relevance is its flexibility. The IDSS needs to be designed to allow adaptation to farm variations but it has to have the right balance between relevance and customization. Relevance is related to how meaningful the IDSS are. Additionally, sustained adoption requires a continuous improvement pipeline addressing both structural things like technology changes related to new ways of collecting, sharing, processing, or storing the data and issues related to the system of production such as new technologies or sensors and new variables available for processing. Innovation speed on sensor development, Internet of Things technologies, and related technologies are advancing at an accelerated pace and IDSS should be able to keep up with that pace. 

Some limiting factors of today will likely be less of an issue in the near future and as such IDSS should be prepared to take full advantage of these upcoming developments. Internet speed and reliability might not be an issue in rural areas anymore when 5G and other network communication technologies become widely available even in rural areas and of common use in dairy farms. Additionally, technologies related to infrastructure (e.g., database management or software) would greatly improve and better support data sharing and data integration protocols. Although these and other technological progress would be important in helping the development of IDSS in the future and consequently their adoption, the core issues of adoption, which are not directly solved by technological improvements, would remain. As earlier identified, the value proposition, easiness of use, practical and direct application, and the willingness of data sharing among data generators and recording systems would remain critical factors for IDSS uptake and sustained adoption. As such, attention to these factors should be part of the continuous improvement pipeline of research and development of IDSS.

Overall, there are several steps with multiple layers that need to be considered for the sustained IDSS adoption. In a simplistic and ideal way, we can summarize those as:

(1) Define clearly and concisely the decision-making problem to be addressed and solved. Additionally, define the IDSS value proposition, which will guide the entire process. The relevance of data integration of different sources must be highlighted as a fundamental part of the solution to the problem.
(2) Define the specific target audience and foster continuous interactive processes such as “focus groups” and others to help build and test the envisioned IDSS. Knowing the profile of the target audience will assure the best IDSS user experience. At this stage, it is worth evaluating possible early adopters, which will have a critical role in the adoption process.
(3) Secure data availability needed for the IDSS, which implies data access, data sharing, data cleaning and data processing in an efficient way, but still maintaining all security protocols needed to guarantee a fair and efficient data exchange and processing.
(4) Nurture resilience and plasticity of the data availability and the IDSS, which may play an increasingly important role for the long term sustainable adoption.

## 5. Conclusions

The challenges associated with the sustainable adoption of IDSS are complex, multifactorial and go beyond those of existing tools because IDSS require a live continuous flow of an integrated stream of multiple data sources. These challenges exist on the farm (i.e., structure for data collection and data processing, financial resources, willingness of the farmer to make data-driven decisions, etc.), in the IDSS (i.e., good value proposition, insightful outcomes, user friendliness, affordable cost, etc.), and in the relevant social interactions and effective cooperation and communication between the main stakeholders involved in the whole development and adoption process. There are, as of yet, few examples of IDSS in the dairy industry, and no successful business models for IDSS. The sustainable adoption of IDSS will depend on the willingness of dairy sector stakeholders to coordinate data sharing that benefits the entire value chain, not only the parts of direct interest to individual links in the chain. The development and sustainable adoption of IDSS would not only directly impact how dairy farmers make decisions within the farm, but potentially reach a broader spectrum of the dairy sector including farmer cooperatives, consultants, processors, retailers and the public. For instance, IDSS could serve to facilitate or even monitor any type of certification process in which the farmer might be interested to be part of. Some examples are greenhouse gas emissions, carbon and energy footprint, animal health and welfare, blockchain, or fair trade certification processes. Adoption of IDSS could also become an important component for decisions made by policy makers. Innovative IDSS will change the way that dairy farms operate in the future and reshape the entire dairy products supply chain. 

## Data Availability

Not applicable.

## USDA Co-Author Disclaimers

The U.S. Department of Agriculture (USDA) prohibits discrimination in all its programs and activities on the basis of race, color, national origin, age, disability, and where applicable, sex, marital status, familial status, parental status, religion, sexual orientation, genetic information, political beliefs, reprisal, or because all or part of an individual’s income is derived from any public assistance program. (Not all prohibited bases apply to all programs.) Persons with disabilities who require alternative means for communication of program information (Braille, large print, audiotape, etc.) should contact USDA’s TARGET Center at (202) 720-2600 (voice and TDD). To file a complaint of discrimination, write to USDA, Director, Office of Civil Rights, 1400 Independence Avenue, S.W., Washington, D.C. 20250-9410, or call (800) 795-3272 (voice) or (202) 720-6382 (TDD). USDA is an equal opportunity provider and employer. Mention of trade names or commercial products in this article is solely for the purpose of providing specific information and does not imply recommendation or endorsement by the U.S. Department of Agriculture. The findings and conclusions in this publication are those of the author(s) and should not be construed to represent any official USDA or U.S. Government determination or policy.
